# The Toxic Effects of Aflatoxin B1 and Aflatoxin M1 on Kidney through Regulating L-Proline and Downstream Apoptosis

**DOI:** 10.1155/2018/9074861

**Published:** 2018-08-12

**Authors:** Huiying Li, Lei Xing, Muchen Zhang, Jiaqi Wang, Nan Zheng

**Affiliations:** Institute of Animal Sciences, Chinese Academy of Agricultural Sciences, Beijing 100193, China

## Abstract

The toxic effects and potential mechanisms of aflatoxin B1 (AFB1), aflatoxin M1 (AFM1), and AFB1+AFM1 in the kidney were studied and compared in HEK 293 cells model and CD-1 mice model. The 35-day subacute toxicity mice model was constructed, biochemical indicators and kidney pathological staining were detected, kidney metabonomics detection was performed, and the metabolites were analyzed, and then the related toxicity mechanism was validated. Results showed that AFB1 (0.5 mg/kg), AFM1 (3.5 mg/kg), and AFB1 (0.5 mg/kg)+AFM1 (3.5 mg/kg) activated oxidative stress and caused renal damage. The relative concentration of the metabolite L-proline was found to be lower in aflatoxins treatment groups when compared with the control (*P* < 0.05). Moreover, with the treatment of aflatoxins, proline dehydrogenase (PRODH) and proapoptotic factors (Bax, Caspase-3) were upregulated, while the inhibitor of apoptosis Bcl-2 was downregulated, at both the mRNA and the protein levels, comparing with the control (*P* < 0.05). In addition, the combined effect of AFB1 and AFM1 was validated, for the toxicity of the combination was stronger than the other two groups. In conclusion, AFB1 and AFM1 caused kidney toxicity by activating oxidative stress through altering expression of PRODH and L-proline levels, which then induced downstream apoptosis.

## 1. Introduction

**Figure 1:**
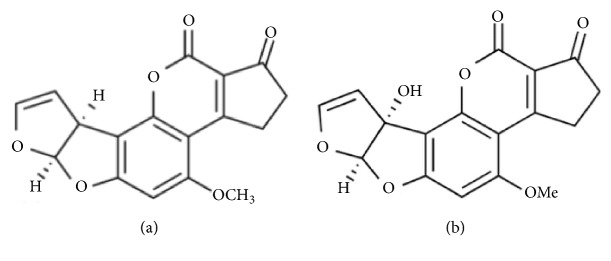
**Chemical structure of AFB1 and AFM1. **(a) Structure of AFB1. (b) Structure of AFM1.

Aflatoxins (AFs) are secondary metabolites with high toxicity synthesized by* Aspergillus flavus* and* Aspergillus parasiticus* which are produced in various feedstuffs, including corn, cottonseed, peanuts, etc. [1, 2]. Aflatoxin B1 (AFB1, Figure 1(a)) is the most common and carcinogenic member in AF family, and IARC (International Agency for Research on Cancer) organization suggested that AFB1 should be classified to be a Group I carcinogen [3]. Another important member of the AF family, aflatoxin M1 (AFM1, Figure 1(b)), is the 4-hydroxy derivative of AFB1, and it can be derived from AFB1 in the liver by the action of hepatic microsomal cytochrome P450, from where it can enter the mammalian circulation and be excreted into the milk of animals in lactation [4–10]. AFM1 is also considered to be carcinogenic and harmful to humans (Group I, IARC) [11].

Apart from being a hazard for the poultry industries and domestic animals, AFs are a critical public health threat, being responsible for Reyes syndrome and acute and chronic liver failure [12]. The metabolic and toxic effects of AFB1 and AFM1 are principally observed in the liver [13–15]; the lung might also be a target organ after exposures of inhalation and diet, and indeed evidences from both laboratory and epidemiologic study validate a carcinogenic effect of AFB1 on human lung tissue [16, 17]. Moreover, several epidemiological studies implicate that AFB1 plays a role in the clinical incidence of gastroenteric tumors of Asians and Africans [18, 19]. Aflatoxins, especially AFB1 and AFM1, are also a pathogenic factor in child underweight, hypoimmunity, neurologic damage, and even high mortality [20]. However, studies investigating the effects of AFB1 or AFM1 on kidney function and revealing the related mechanism are rare.

It is reported that excretion of AFB1 and AFM1 occurs primarily through the biliary pathway, followed by the urinary pathway, and AFB1 could be detected in different levels in the kidney and urine of two calves with dosages of 0.8 mg and 1.8 mg /kg body weight, respectively [21]. However, the mechanism of the toxicity of the two AFs and their metabolites is still unclear.

Direct evidence for the exposure of humans to AFs by ingestion or another route has been found in a number of countries by identifying AFs or their metabolites in human biological samples [22–24]. Thus, it is becoming a pressing and necessary issue, not only for healthy adults, but also for nurslings, who are being directly exposed to food contaminated with AFs. Therefore, further research into the mechanism of AF toxicity is now necessary and urgent. The purpose of the present research was to investigate the toxic effects of AFB1 and AFM1 on the kidney tissue, and especially of the combination of the two, and to explore the mechanisms involved.

## 2. Materials and Methods

### 2.1. Chemicals and Reagents

Ninety-five percent pure AFB1 and AFM1 were purchased from Pribolab (Singapore). HEK 293 cells (a human epithelial kidney cell line) was obtained from the American Type Culture Collection Cells (ATCC, USA). Dulbecco's Modified Eagle Medium (DMEM) and fetal bovine serum (FBS) were purchased from GIBCO (USA), L-glutamine was purchased from ChemCatch (USA), and 1% penicillin/streptomycin was purchased from Thermo Fisher (USA). Cell counting kit-8 (CCK-8 kit) was purchased from Dojindo (Japan). ELISA detection kits for creatinine (Scr), urea (UREA), uric acid (UA), malondialdehyde (MDA), superoxide dismutase (SOD), and total antioxidant capacity (T-AOC) in mouse serum were purchased from Jiancheng (Nanjing, China). A hematoxylin-eosin (HE) staining kit, a total protein extraction kit, and TBST buffer were purchased from Solarbio, (Beijing, China). *β*-actin, proline dehydrogenase (PRODH), Δ1-pyrroline-5-carboxylate synthetase (P5CS), Δ1-pyrroline-5-carboxylate reductase (P5CR), B-cell lymphoma-2 (Bcl-2), Bcl-2 Associated X Protein (Bax), and cysteinyl aspartate specific proteinase 3 (Caspase-3) antibodies, and secondary antibody were purchased from Santa Cruz (USA), and ECL reagent was purchased from Thermo Fisher (USA).

### 2.2. Cell Culture and Viability Detection

HEK 293 cells were cultured with DMEM,10% FBS, 0.9% L-glutamine, and 1% penicillin/streptomycin, in a humidified incubator (Thermo, USA) at 37°C, in the presence of 5% CO_2_. They were exposed to AFB1, AFM1, or AFB1 + AFM1 at various concentrations (0-200 mg/L) and cocultured for 48 h; then the cell viability was quantified using a CCK-8 kit (Solarbio, Beijing) to choose the most appropriate dose for further experiments.

### 2.3. Animal Model

CD-1 mice were purchased from Beijing Vital River Laboratory Animal Technology Co., Ltd. (Beijing, China), with license number SCXK 2012-0001. Animals were fed in cages at 25°C with a relative humidity of 55%. The mice were acclimatized for at least seven days before commencement. All animal procedures were performed according to the Chinese guidelines for animal care, conforming to internationally accepted principles for the care and utilization of experimental animals. Animal experiments were approved by the Ethics Committee of the Chinese Academy of Agricultural Sciences (Beijing, China).

Thirty-two CD-1 mice (20 ± 2 g, male) were randomly divided into four groups: a control group (untreated), a 0.5 g/kg AFB1 group, a 3.5 g/kg AFM1 group, and a combination group (0.5 g/kg AFB1 + 3.5 g/kg AFM1), containing eight mice per group. AFB1 and AFM1 were dissolved in DMSO/ddH_2_O (final 1%/99%) [25]. The mice in the treatment groups were gavaged once per day (0.2 mL/ mice) for 28 days and then sacrificed on day 29. Blood samples were gathered from the retro-orbital plexus, and the kidney was dissected and frozen in liquid nitrogen for subsequent metabonomic analysis and histopathology.

One hundred milligrams of kidney sample was added to 1 mL tubes and incubated in 1 mL 50% methanol for 5 min, until they had completely dissolved. The suspensions were then centrifuged at 10,000 rpm for 10 min. The upper layer (800 *μ*L) was collected into new 2 mL glass tubes and 5 *μ*L sample was analyzed using a UHPLC Q-Orbitrap, triplicate measurements in each aliquots.

### 2.4. Histopathological Test

Kidney tissue was isolated and fixed in 4% paraformaldehyde for 48 h, before paraffin embedding and sectioning using a microtome (Leica, Germany). The sections were stained with HE and the histopathology assessed under a light microscope (Olympus, Japan), with photographs being taken at 200 × magnification.

### 2.5. Biochemical Analysis

Retro-orbital blood samples were centrifuged to collect serum (15 min at 3,000 rpm and 4°C) for the measurement of biochemical parameters, including creatinine (Scr), urea (UREA), uric acid (UA), malondialdehyde (MDA), superoxide dismutase (SOD), and total antioxidant capacity (T-AOC), which was undertaken using ELISA kits (Jiancheng, Nanjing).

### 2.6. Tissue Metabonomics Detection and Data Mining

Metabolites were separated in a UHPLC system (Dionex Ultimate 3000) equipped with a Waters Column (Acquity BEH C18 1.7 *μ*m, 2.1 × 50 mm) at 40°C. The mobile phase consisted of water containing 0.1% formic acid and 2 mM ammonium formate (solvent a, v/v), and acetonitrile containing 0.1% formic acid and 2 mM ammonium formate (solvent b, v/v), with a flow rate of 250 *μ*L/min, and the following gradient elution program: 0–1.0 min, 5% b; 1.0–5.0 min, 5% to 60% b; 5.0–8.0 min, 60% to 100% b; 8.0–11.0 min, 100% b; 11.0–14.0 min, 100% to 60% b; 14.0–15.0 min, 60% to 5% b; 15.0–18.0 min, 5% b. The Q-Exactive instrument (Thermo) equipped with electrospray ionization in positive and negative switching modes was utilized to detect the above samples, and the system was calibrated and controlled by Xcalibur 3.1 and Q-Exactive Tune software. The UHPLC Q-Orbitrap analysis can produce large amounts of raw data using TraceFinder software. The data was exported into Excel spreadsheets by Simca-P for PCA (principle components analysis), PLS-DA (partial least squares discriminant analysis), t-test, volcano plot, and VIP (variable importance in projection) plot analysis [26].

### 2.7. Quantitative Real-Time PCR Analysis

Fifty to two hundred nanograms of total RNA was extracted from mouse kidney using a TransZol Up Kit (ET111-01, TransGen Biotech, Beijing, China). The quantity and concentration of RNA were evaluated by 1.2% agarose gel electrophoresis and Nanodrop 2000 (Thermo Fisher, USA). The total RNA was transcribed into cDNA utilizing a High Capacity cDNA Archive Kit (Applied Biosystems, CA). Primers for the evaluated genes are listed in Table 1. Quantitative real-time RT-PCR (qRT-PCR) was performed in 96-well plates (0.5 *μ*L (10 *μ*M) forward primer, 0.5 *μ*L (10 *μ*M) reverse primer, 1 *μ*L template cDNA (cDNA, 10–15ng/*μ*L), 10 *μ*L Universal Master Mix, and 8 *μ*L of RNAse-free water), all reagents were obtained from Applied Biosystems by Life Technologies, USA. All qRT-PCR reactions were performed at 94°C for 30 s, followed by 40 cycles of 94°C for 5 s, and 62°C for 30 s, using two-step RT-PCR. All qRT-PCR reactions were performed on the ABI 7900 HT system and were conducted in triplicate to ensure methodological reproducibility.

### 2.8. Western Blotting Analysis

Total protein in cells or kidney tissue was extracted by a protein extraction kit (Solarbio, Beijing, China). After catalysis and heat treatment, the protein samples were separated on 12% SDS-polyacrylamide gels, the proteins were transferred onto nitrocellulose membranes with Trans-Blot machines (Bio-Rad), and the membranes were blocked with 2% BSA in TBST buffer for 1 h at 25°C. The membranes were then incubated with primary antibodies at 4°C overnight (targeting *β*-actin, PRODH, P5CR, P5CS, Bcl-2, Bax, and Caspase-3), with *β*-actin being used as an internal reference to confirm equal loading. After three washes with PBST buffer (15 min × 3), the membranes were incubated with secondary antibodies at 37°C for 1 h and then washed (15 min × 3). Finally, specific protein bands were detected using ECL reagent and analyzed using Image J software [27, 28].

### 2.9. Statistical Analysis

All the data are presented as mean ± SD. Data analysis was performed using GraphPad Prism 6.0 software (GraphPad, San Diego, USA). Statistical analysis was conducted using Student's t-test and One-Way Analysis of variance (ANOVA). *P* < 0.05 was considered to indicate a statistically significant difference between the control and treatment groups.

## 3. Results

### 3.1. AFB1, AFM1, and AFB1 + AFM1 Inhibit Viability of HEK 293 Cells

**Figure 2:**
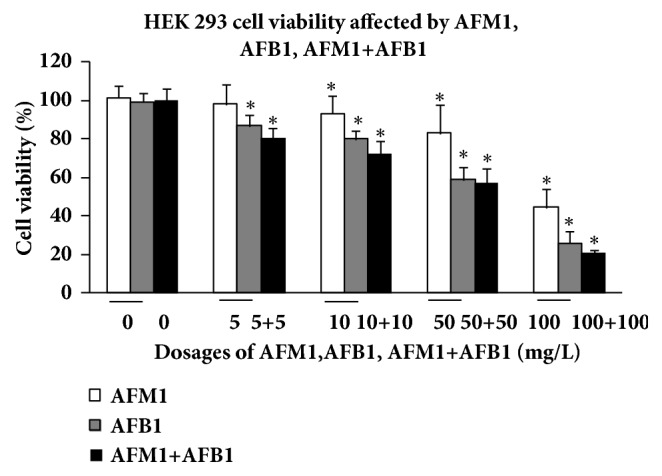
**Comparison of HEK 293 cell viability affected by AFM1, AFB1, and AFM1+AFB1**. The viability rate was represented as mean ± SD, ^*∗*^*P* < 0.05, compared with control (n=8), ^#^*P* < 0.05, compared with control (n=8).

To investigate the effects of AFB1, AFM1, and AFB1 + AFM1 on the kidney cells, human embryonic kidney 293 (HEK 293) cells were utilized and viability of HEK 293 cells was detected using CCK-8 kit. At the same concentration, AFB1 showed a stronger inhibitory effect (26% in the 100 mg/L group) on cell viability than AFM1 (44% in the 100 mg/L group), both of which were within the linear dose-effect range. The combination of AFB1 and AFM1 also inhibited HEK 293 cell viability (21% in the 100 + 100 mg/L group), with a steeper dose-effect relationship than the other two groups (*P* < 0.05), suggesting that the combination of two aflatoxins with high dosage (100 mg/L or above) showed an enhanced suppressed effect on HEK 293 cells when compared with the single aflatoxin treatment group (Figure 2).

### 3.2. AFB1, AFM1, and AFB1 + AFM1 Affect Serum Biochemistry Indicators

**Figure 3:**
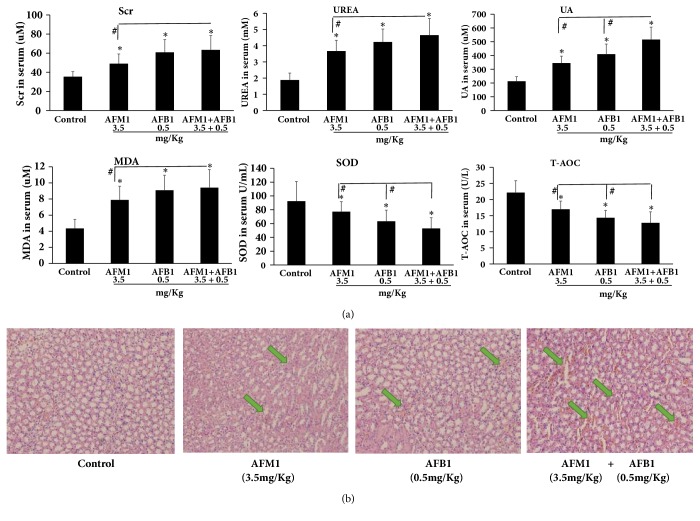
**Kidney damage caused by AFM1, AFB1, and AFM1+AFB1: biochemical indicators in serum and kidney tissue HE staining. **(a) Scr, UREA, UA, MDA, SOD, and T-AOC detection. The values of biochemical indicators were represented as mean ± SD, ^*∗*^*P* < 0.05, compared with control groups; ^#^*P* < 0.05, compared with AFM1 treatment group (n=8). (b) Kidney tissue pathological detection by pathological staining with hematoxylin and eosin. The pathological pictures were captured at 200 × magnification, and the blue arrows showed the injury area.

To evaluate the effect of the AFs on kidney function, mouse serum was collected and three markers were measured by ELISA. Twenty-eight days of AFB1, AFM1, or AFB1 + AFM1 administration caused sharp increases in creatinine (Scr), urea (UREA), and uric acid (UA) (all* P* < 0.05 versus control). To investigate the effect of AFs on oxidative stress, malondialdehyde (MDA), superoxide dismutase (SOD), and total antioxidant capacity (T-AOC) were measured in mouse serum; results revealed that MDA was upregulated and markedly higher, and SOD and T-AOC decreased and were significantly lower, when compared with the control group (*P* < 0.05). Furthermore, UA, SOD, and T-AOC in the AFB1 + AFM1 group changed significantly compared to the ones in the other two groups (*P *< 0.05), and Scr, UREA, and MDA in the AFB1 + AFM1 group increased significantly compared to the ones in the other two groups (*P* < 0.05) (Figure 3(a)).

### 3.3. AFB1, AFM1, and AFB1 + AFM1 Induce Kidney Pathology

To further investigate the effects of AFs on kidney, HE staining of histological sections was performed; results showed that aflatoxins caused obvious injury in kidney tissue. Compared with the control group, some areas of sections in AFB1-treated and AFB1 + AFM1-treated groups demonstrated edema and cytomorphosis, and occasional severe inflammatory cell infiltration and hemorrhage, whereas the renal injury induced by AFM1 was less severe (Figure 3(b)).

### 3.4. Metabonomics Analysis

**Figure 4:**
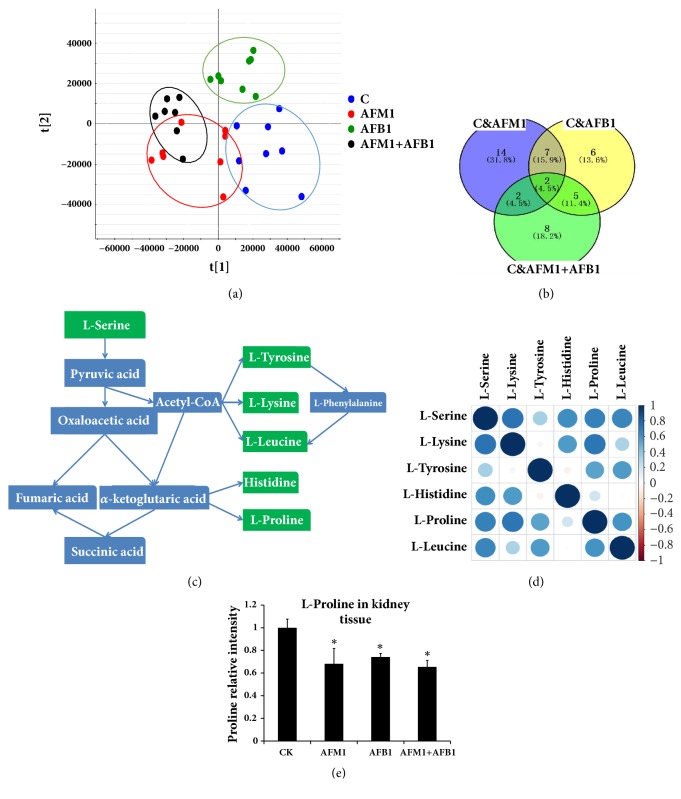
**Tissue metabonomics detection and different metabolites analysis.** (a) OPLS-DA scores plot. (b) VENN plot. (c) Amino acid metabolic pathway. (d) Relation spot. The depth of colours and the size of the circle stand for value of the correlation: the darker the colour, the bigger the circle; the correlation index was higher. (e) L-proline detection in kidney tissue by mass spectrum. All the data was represented as mean ± SD, ^*∗*^*P* < 0.05, compared with the control (CK) (n=8).

To evaluate the effect of the two aflatoxins on kidney metabolism, the metabonomic detection of kidney tissue from mice treated with AFB1, AFM1, or AFB1 + AFM1 was performed. The metabolites clustering in the AF-treated groups was clearly different from those in the control (Figure 4(a), OPLS-DA scores plot), indicating that sample treatment and data analysis were stable and valid. By comparing the levels of metabolites among the four groups, twenty-five metabolites were found to be changed significantly in AFM1 treatment group when compared with the control, twenty metabolites were expressed significantly in AFB1 treatment group when compared with the control, and seventeen metabolites were screened out in AFM1 group comparing with the AFB1. Finally, two metabolites with VIP value ≥ 1 (L-proline and creatinine) were identified by overlapping three parts of the above results (Figure 4(b), VENN diagram).

In addition to L-proline, L-serine, L-lysine, L-tyrosine, L-histidine, and L-leucine were all shown in the overlapping area between the AFM1 and AFB1 groups, and the relevant metabolic pathway and relation spot were shown in Figures 4(c) and 4(d). The concentrations of these amino acids were lower in the three aflatoxin treatment groups when compared with the control. The level of L-proline in the four groups was measured by mass spectrometry; results showed that L-proline concentration was lower in AF-treated samples (*P* < 0.05) than the control level, and there were no obvious differences between the AFB1 + AFM1 group and the other two groups (Figure 4(e)).

### 3.5. Toxic Effects of AFB1, AFM1, and AFB1 + AFM1 on Kidney via PRODH

**Figure 5:**
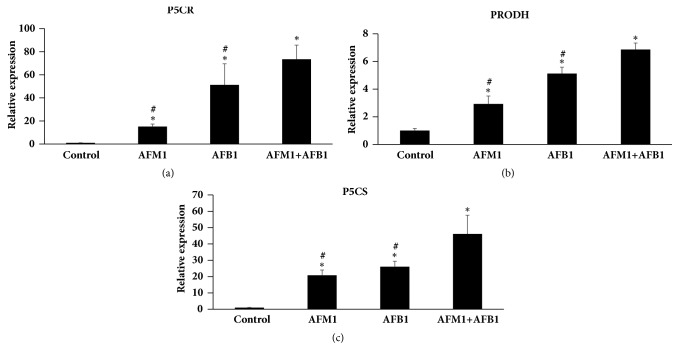
**Detection of PRODH/P5CR/P5CS in kidney tissue by q-PCR.** (a) The level of P5CR. (b) The level of PRODH. (c) The level of P5CS. All the value was represented as mean ± SD, ^*∗*^*P* < 0.05, compared with control; ^#^*P* < 0.05, compared with the one in (AFB1+AFM1) group (n=8).

**Figure 6:**
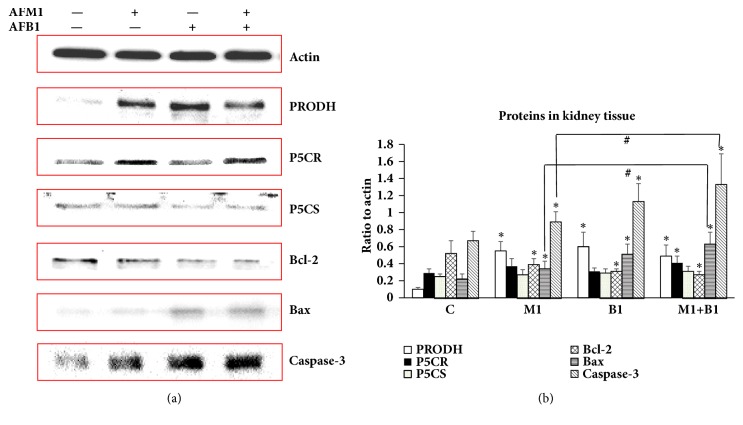
**Detection of PRODH/P5CR/P5CS/Bcl-2/Bax/Caspase-3 in kidney tissue by western blotting.** (a) Gray bands of PRODH/P5CR/P5CS/Bcl-2/Bax/Caspase-3 in western blotting. (b) Quantification of expression levels of PRODH/P5CR/P5CS/Bcl-2/Bax/Caspase-3 by Image J Software. All the values were represented as mean ± SD, ^*∗*^*P* < 0.05, compared with control; ^#^*P* < 0.05, compared with the one in (AFB1+AFM1) group (n=8).

To further investigate the toxic mechanism of the two AFs and to validate the relationship between L-proline and AFB1/AFM1, the expressions of P5CR, P5CS, and PRODH were measured in kidney. q-PCR analysis showed that mRNA expressions of all three factors were higher in the AF-treatment groups than the control (*P* < 0.05), and their expressions in the combined treatment group were much higher than in the other two aflatoxins treatment groups (*P* < 0.05) (Figure 5). Results of western blotting detection showed that addition of AFB1, AFM1, or AFB1 + AFM1 also significantly increased protein levels of PRODH, Bcl-2, Bax, and Caspase-3 (*P* < 0.05), while expressions of P5CR and P5CS were not affected, suggesting that PRODH might be the target of the AFs (Figure 6).

**Figure 7:**
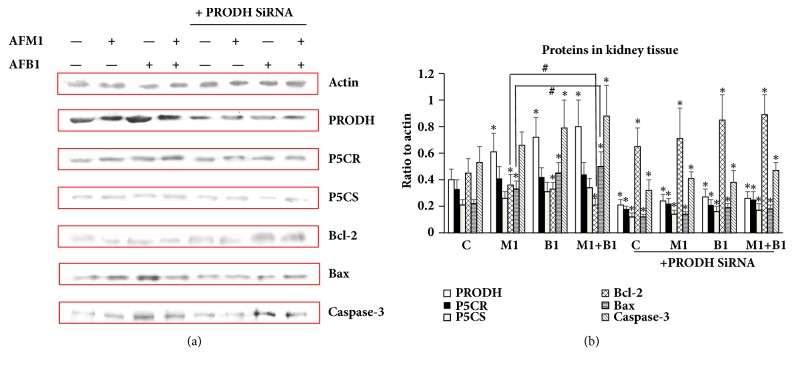
**Detection of PRODH/P5CR/P5CS/Bcl-2/Bax/Caspase-3 in HEK 293 cells by western blotting.** (a) Gray bands of PRODH/P5CR/P5CS/Bcl-2/Bax/Caspase-3 in western blotting. (b) Quantification of expression levels of PRODH/P5CR/P5CS/Bcl-2/Bax/Caspase-3 by Image J Software. All the values were represented as mean ± SD, ^*∗*^*P* < 0.05, compared with control; ^#^*P* < 0.05, compared with the one in (AFB1+AFM1) group (n=8).

After transfection with PRODH siRNA, the levels of these proteins were markedly lower (*P* < 0.05) than in the control cells, and when AFs were then added to the cells, the expression of the above proteins did not increase significantly compared to the PRODH siRNA group (Figure 7). These data indicate that PRODH is a direct target of AFs, and that it is responsible for activating downstream apoptosis pathways.

## 4. Discussion

Based on the biochemical measurements demonstrating the presence of higher concentrations of Scr, UREA, and UA, long-term administration of AFs was shown to cause renal damage, which might involve inflammation, cell necrosis, and toxicosis [29–39]. Our histological findings were consistent with renal injury caused by AFs and were in accordance with the biochemical data. Together, these results confirmed that the kidney was one of the main target organs of AFs and indicate that several metabolites might be transferred, produced, or degraded in the kidney, such as proline, which was validated to be a special metabolite in kidney in the present study.

AFs are potent carcinogenic and genotoxic compounds, which exert toxic effects through DNA damage and mutations leading to oxidative damage. With regard to the mechanism of oxidative damage caused by AFs, cell inactivation by proteasomes was regarded as a part of the cellular defense against oxidative stress, and AFB1 and AFM1 were reported to be the most potent activators of proteasome activity [40–43]. In the current study, malondialdehyde (MDA), superoxide dismutase (SOD), and total antioxidant capacity (T-AOC) in mouse serum were measured by ELISA, and it was found that MDA was markedly higher, and that SOD and T-AOC were much lower in AF-treated mice. As a peroxide produced by free radicals, MDA tissue content reflects the degree of oxidative damage [44]. In our study, aflatoxins induced oxidative stress as evidenced by peroxidation of lipids and MDA in the serum. SOD is a classical antioxidant enzyme in various organisms which converts superoxide anion radicals to hydrogen peroxide and protects organisms from oxidative injury. T-AOC reflects the activity of all the antioxidants in an organism and thus is an indicator of overall antioxidative activity [45, 46]. Aflatoxins in our model resulted in releasing free radicals especially superoxide anions in kidney tissue; lots of T-AOC factors including SOD in serum were recruited into the tissue, resulting in downregulation of T-AOC and SOD in serum. The effects of AFs on these parameters are thus consistent with their activation of oxidative reactions in the mice.

Proline is a metabolite of AFB1 and AFM1, and we found that its concentration was significantly lower in mice treated with these AFs than the control mice. Previous studies suggest that, in addition to providing energy, metabolism of proline affects oxidative stress in various organisms [47–53]. Rai et al. found that proline alleviates damage from reactive oxygen species, rather than improving the antioxidant defense system, when cells are placed under metal stress [54]. Krishnan et al. also reported that proline protected cells against H_2_O_2_, tert-butyl hydroperoxide, and a carcinogenic oxidative stress inducer [47]. It is reported that, in Gram-negative bacteria, the proline utilization A flavoenzyme containing PRODH and P5C dehydrogenase domains in a single polypeptide could catalyze the oxidation of L-proline to glutamate [55]. Therefore, proline improves oxidative stress tolerance in* E. coli* by a preadaptive effect involving the production of endogenous hydrogen peroxide and the enhancement of catalase-peroxidase bioactivity [52]. Here, PRODH activity was shown to increase in response to AFs, along with caspase and Bax, suggesting induction of apoptotic cell death.

In our study, PRODH mRNA and protein were significantly higher in AF-treated mice, and cell apoptosis in kidney tissue was also significantly activated, embodied on changing expressions of Bcl-2, Bax, and Caspase-3. Proline concentrations were lower in kidney tissue from AF-treated mice, which was likely the result of PRODH upregulation. PRODH siRNA treatment was used to determine whether PRODH is the direct target of AFs, and we found that the expression of P5CS, P5CR, and proapoptotic factors was no different in AF-treated and siRNA-treated cells, and in cells that were treated with PRODH siRNA alone. These findings confirm that the two AFs activate oxidative reactions and have a toxic effect on mouse kidney, mainly through a reduction in the level of their metabolite, proline, which is regulated by PRODH. In addition, we have shown that downstream apoptotic factors including Bcl-2, Bax, and Caspase-3 are impacted by PRODH siRNA treatment.

Previous research showed that AFB1 and AFM1 acted synergistically with hepatitis B virus (HBV), which resulted in the increased risk of liver carcinoma by 12-fold, while Zhang H. demonstrated similar effects of AFB1 and AFM1 in various cell types [56–58]. Then we found that the sequence of overall toxicity and oxidative damage degree of AF-treated groups was AFB1+AFM1 > AFB1 > AFM1, suggesting that the two aflatoxins might act synergistically, which deserved more attention and data in the further research.

**Figure 8:**
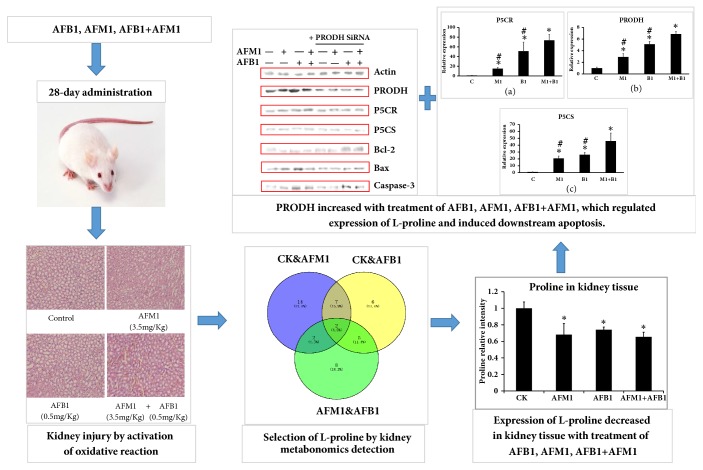
**TOC Graphic. **With the 28-day administration of AFB1, AFM1, and AFB1+AFM1, mice serum biochemical indicators changed significantly; mice kidney tissue showed injury with different degrees, when compared with the control, indicating AFB1, AFM1, and AFB1+AFM1 caused kidney damage and activated oxidative reaction. Through metabonomics detection of kidney tissue, L-proline was verified to be the key metabolite in kidney tissue in response to these aflatoxins. Then in mechanism exploration part, PRODH was validated to be the upstream regulator of L-proline, which also induced downstream apoptosis.

In summary, we have identified a key metabolite of AFB1 and AFM1 treatment, L-proline, by metabonomic screening of liver extracts from mice treated with either AF or a combination. We have also shed light on the effect of the upstream sensor PRODH, which regulates the level of L-proline, leading to kidney damage through the induction of oxidative stress and apoptosis. As Figure 8 (TOC Graphic) showed, L-proline will be utilized to detoxify kidney damage caused by aflatoxins in mice model, and the related mechanism and specific action sites will be revealed and validated through transcriptomics detection and bioinformatic analysis. These findings improve our understanding of the risks associated with the ingestion of AFs and their metabolites and imply that the guidelines for food safety evaluation and AF limits in standard formulations should be modified accordingly.

## Figures and Tables

**Table 1:** Primers of the genes.

Gene Name	Primer Sequences (5' → 3')	
	Forward Primer	Reverse Primer
P5CR	ATGTGCTCTTCCTGGCTGTGA	GCGTGAGTACCTGTGGCATAC
PRODH	CGTGGACTTGCTGGACTGGAA	CGGCTGATGGCTGGTTGGAA
P5CS	ATCATCTGGCTGACCTGCTGAC	GTGAAGAATGCGGTTGCTGTGT
GAPDH	CGTCCCGTAGACAAAATGGT	TTGATGGCAACAATCTCCAC

## Data Availability

The data used to support the findings of this study are included within the article.

## Abbreviations

AFB1: Aflatoxin B1
AFM 1: Aflatoxin M1
PRODH: Proline dehydrogenase
P5CS: Δ1-Pyrroline-5-carboxylate synthetase
P5CR: Δ1-Pyrroline-5-carboxylate reductase
Bcl-2: B-Cell lymphoma-2
Bax: Bcl-2 Associated X Protein
Caspase-3: Cysteinyl aspartate specific proteinase 3.
